# Blood parasites and bacteria of Muroidea rodents of the Chornobyl Exclusion Zone

**DOI:** 10.1016/j.ijppaw.2026.101250

**Published:** 2026-06-12

**Authors:** Vladyslava Storozhuk, Hans-Peter Fuehrer, Bita Shahi Barogh, Barbara Eigner, Alla Lypska, Olena Burdo, Denys Vyshnevskyi, Olena Semenko, Caroline F. Frey, Maryna Galat

**Affiliations:** a Faculty of Veterinary Medicine, National University of Life and Environmental Sciences of Ukraine, Heroiv Oborony Str. 15, Kyiv, 03041, Ukraine; b Parasitology, Department of Biological Sciences and Pathobiology, University of Veterinary Medicine Vienna, Veterinärplatz 1, Vienna, 1210, Austria; c Institute for Nuclear Research of the National Academy of Sciences in Ukraine, Prospekt Nauky Str. 47, Kyiv, 03680, Ukraine; d Chornobyl Radiation and Ecological Biosphere Reserve, Chornobyl, Ukraine; e Faculty of Veterinary Medicine, University of Alfonso X, Campus Villanueva, University Av., 1, Villanueva da la Cañada, Madrid, 28691, Spain; f Institute of Parasitology, Department of Infectious Diseases and Pathobiology, Vetsuisse Faculty, University of Bern, Länggassstrasse 122, Bern, CH-3012, Switzerland

**Keywords:** Rodents, Radiation, *Trypanosoma*, *Hepatozoon*, Mollicutes, *Bartonella*, Microscopy, PCR, Sequencing, Ukraine

## Abstract

The Chornobyl Exclusion Zone (ChEZ) is a unique area in the world. It combines two things: the largest man-made disaster site in the world and a radioecological observatory. The observatory has been studying the long-term impact of radioactive contamination on the environment, flora and fauna since 1986. Muridae and Cricetidae rodents being extremely widespread representatives of ChEZ fauna, serve as bioindicators for radioecological research.

During 2019-2020, in addition to studying rodents as markers of radioactive contamination, 116 muroids were examined for the presence of parasites and bacteria. Among the rodents studied were bank voles *Myodes glareolus* from the Cricetidae family, as well as yellow-necked mice *Apodemus (Sylvaemus) flavicollis* and striped field mice *Apodemus agrarius* of the Muridae family.

Microscopy of muroid rodent blood films revealed the presence of *Trypanosoma* spp., *Hepatozoon* spp. and bacterial agents. Molecular analyses led to the first identification of *Trypanosoma grosi* in *A*. *agrarius* in Ukraine. A *Hepatozoon* spp. SK3-type was found for the first time in *A*. *flavicollis.* Three different genotypes of Mollicutes were detected in *A*. *agrarius* and *A. flavicollis*. *Bartonella* spp. were present in most of the rodents tested. Zoonotic *Bartonella grahamii* was identified among six isolated genotypes of *Bartonella*. Co-infections with parasites and bacteria were found in some animals.

According to microscopic studies, *A. agrarius* had the highest prevalence of *Trypanosoma* spp., whereas *Hepatozoon* spp. was highest in *M. glareolus*. Meanwhile, *A*. *flavicollis* harboured the greatest diversity of genotypes according to the sequencing results. Interestingly, a 41.7% prevalence of *T*. *grosi* and 33.3% of *Mycoplasma*-like bacteria was detected by PCR among animals from landfill II of the ChEZ, which was the least contaminated of the four experimental sites in the Exclusion Zone.

Further research should be conducted to identify pathogens of viral origin and to study more genome loci of parasites in order to determine the impact of radioactive contamination that microorganisms may be exposed to.

## Highlights

• Prevalence of parasites and bacteria in rodents of the ChEZ was studied.
• Trypanosomes found in *Apodemus agrarius* have been identified as *Trypanosoma grosi.*
• Genotypes of *Hepatozoon* spp., Mollicutes and *Bartonella* spp. were identified.

## Graphical abstract

**Figure:**
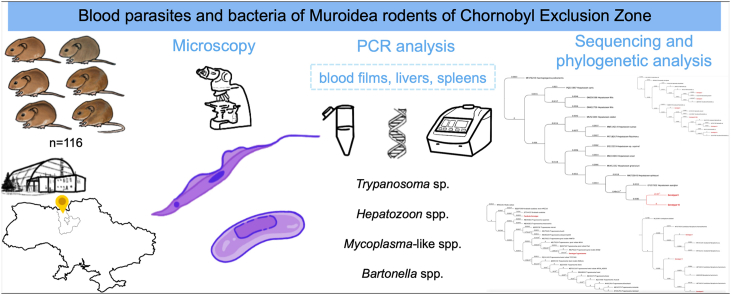

## 1 Introduction

The Ukrainian part of the Chornobyl Exclusion Zone (ChEZ) stretches for approximately 2598 km^2^ and is a unique territory without human activity for about 40 years due to the world's largest man-made disaster. This territory serves as radioecological observatory for long-term studies on the impact of radioactive contamination on the environment (Rodgers et al., 2001; Wickliffe et al., 2002; Tsyusko et al., 2006; Napier et al., 2007; Steiner et al., 2013; Kashparov et al., 2018; Wood et al., 2023; Boratyński et al., 2025). The ChEZ spans forests, wetlands, flowing and standing water bodies, as well as abandoned agricultural land, villages and urban areas. Therefore, the absence of anthropogenic pressure, the high heterogeneity of habitats, and the abundant and relatively untouched food resources on this territory, even despite radioactive contamination, are favourable for the spread of rodents. Muroidea rodents (order Rodentia, suborder Myomorpha, superfamily Muroidea) (muroids) belong to the largest and species-rich superfamily of mammals (Steppan et al., 2004; Steppan and Schenk, 2017). Being widespread representatives of ChEZ fauna, they serve as bioindicators for radioecological research. Muroid rodents are exposed to both internal and external effects of radioactive contamination. External exposure includes contact with soil contaminated ^137^Cs, ^90^Sr, and other isotopes, as well as living in burrows, which can sometimes be as deep as 20 cm. Observations by Gaschak et al. (2011) indicate that the level of radioactive contamination of the territories is gradually decreasing. While in 1986, following the accident at the Chornobyl Nuclear Power Plant (ChNPP) that exposed the reactor core, the absorbed dose rate reached 1.3-6.0 Gy h^−1^, by 2005 this level had decreased significantly to 0.00015 Gy h^−1^. Internal exposure includes the rodents' diet and bioavailability of radionuclides in the “soil-to-plant” chain (Burdo et al., 2020). Therefore, rodents are a model for studying radioactive contamination in the context of ChEZ conditions, including for identifying interspecies differences in radionuclide accumulation and the factors that influence it (Chesser et al., 2001; Gaschak et al., 2011; Burdo et al., 2020; Lypska et al., 2022; Boratyński et al., 2025).

The most common in the vast areas of Europe are *Apodemus agrarius* (Pallas, 1771), *Apodemus (Sylvaemus) flavicollis* (Melchior, 1834) and *Apodemus sylvaticus* (Linnaeus, 1758) of the family Muridae (Filippucci et al., 2002; Michaux et al., 2005; Fabbri et al., 2026). They are omnivorous and occur in habitats ranging from open agricultural fields (*A. agrarius*) to mature forest stands (*A. flavicollis*), with *A. sylvaticus* occupying a highly generalist niche in mosaic landscapes (Zub et al., 2012; Dammhahn et al., 2020). Among the cricetid voles (family Cricetidae), a common representative is a bank vole *Myodes glareolus* (Schreber, 1780), which typically predominates in woodland and structurally complex habitats (Zub et al., 2012; Amirpour Haredasht et al., 2013). Representatives of Muridae and Cricetidae families are also known as reservoir hosts for agents of potentially dangerous zoonotic diseases, including those caused by parasites, bacteria and viruses (Yabsley and Shock, 2012; Amirpour Haredasht et al., 2013; Hildebrand et al., 2013; Szewczyk et al., 2021).

Since rodents have, over the last few decades, served as a model for studying the acute and subsequent chronic effects of radioactive exposure on living organisms, arising from extensive and prolonged environmental contamination caused by low-altitude dispersal of core material following nuclear fission and radioactive fallout after the accident at the ChNPP on 26 April 1986 (Imanaka et al., 2015), the aim of our study was to determine the relationship between the level of radioactive contamination (air, soil, animal's tissues) and the prevalence and characteristics of parasites and bacteria in Muroidea rodents from the ChEZ.

## 2 Materials and methods

### 2.1 Muroidea rodent trapping and sampling

During July, September and October 2019 and 2020, 116 muroids that were captured in Kyiv region, including the territories at the landfills of the drained bed of the cooling reservoir of the ChNPP in the ChEZ (Ukraine), were examined. In 2019, 30 rodents were trapped at sites landfill I (n = 11), landfill II (n = 12) and landfill III (n = 7), and all other animals were captured in 2020 at sites landfill II (n = 12), landfill III (n = 12), Rudyi Lis (n = 20), and Chernechyi Lis (n = 42). The species of muroids were identified by morphological characteristics (Zagorodniuk, 2002). Among the species studied were *A. agrarius* (n = 19), *A*. *flavicollis* (n = 49), *A. sylvaticus* (n = 5), *Apodemus uralensis* (Pallas, 1811) (n = 1), *Mus musculus* Linnaeus, 1758 (n = 1) (Muridae: Murinae) and *M*. *glareolus* (Cricetidae: Arvicolinae) (n = 31). The last 10 muroids could not be identified to the species either by morphological characteristics or by barcoding. The unidentified muroids most likely belonged to the genus *Apodemus* (n = 9) and genus *Microtus* (n = 1) and all of them were recorded as unidentified in the Results of this study. The animals were weighed using scales of model XAS 160/X (RADWAG, Poland). The sex of the rodents was determined in accordance with standard procedures (Zagorodniuk, 2002).

At each site, animals were captured using Sherman live traps, which were arranged in a linear transect of 50 traps spaced 4 m apart. In total, 1050 trap-nights were completed. Trapping was conducted over 3 consecutive days, and white bread fried in unrefined sunflower oil was used as bait. Sampling was carried out for the purpose of determining the level of radioactive contamination in accordance with the programme of the Institute for Nuclear Research of the National Academy of Sciences of Ukraine (Kyiv, Ukraine) (Lypska et al., 2022; Riabchenko et al., 2025).

### 2.2 Study sites

**Fig. 1:**
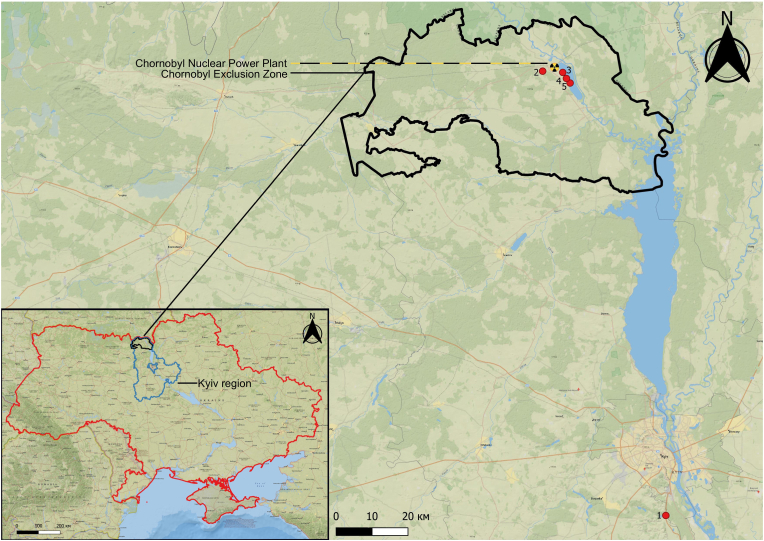
Location of muroid rodents trapping sites in Kyiv region: 1 - Chernechyi Lis (Monk's Forest) between the villages of Khodosivka and Lisnyky (50°17′22.3″ N, 30°31′19.91″ E), 2 - Rudyi Lis (Red Forest) in the south of the abandoned village of Yaniv (51°23′39.0″ N, 30°03′36.0″ E), 3 - landfill I on the coastline of the Chornobyl Nuclear Power Plant cooling pond (51°21′38.6″ N, 30°08′23.50″ E), 4 - landfill II on the drained area of the hot part of the Chornobyl Nuclear Power Plant cooling pond (51°22′20.60″ N, 30°08′26.94″ E), 5 - landfill III on the dam of the first stage of the Chornobyl Nuclear Power Plant cooling pond and the drained hot part of the cooling pond bed (51°21′04.81″ N, 30°09′29.46″ E).

The trapping sites included various habitats: plant communities of pine, mixed and sometimes deciduous forest, marsh and temperate meadow ecotone. In the Rudyi Lis (Red Forest) in the south of the abandoned village of Yaniv (2.7 km to the west from the place of the nuclear reactor explosion, 51°23′39.0″ N, 30°03′36.0″ E) 20 individuals were captured, in landfill I on the coastline of the ChNPP cooling pond (51°21′38.6″ N, 30°08′23.50″ E) 11 individuals, in landfill II on the drained area of the hot part of the ChNPP cooling pond (51°22′20.60″ N, 30°08′26.94″ E) 24 individuals, in landfill III on the dam of the first stage of the ChNPP cooling pond and the drained hot part of the cooling pond bed (51°21′04.81″ N, 30°09′29.46″ E) 19 individuals. All four of these rodent trapping sites are located within ChEZ. An additional sampling site located about 100 km beyond the boundaries of the ChEZ was Chernechyi Lis (Monk's Forest) between the villages of Khodosivka and Lisnyky (50°17′22.3″ N, 30°31′19.9″ E, Kyiv oblast). A total of 42 individuals were captured at this site for comparison with animals originating from the ChEZ. The geographical coordinates of the muroid trapping sites were visualised on the map (Fig. 1) showing the official geographical boundaries of the ChEZ and of Kyiv region using software QGIS (version 3.40 BRATISLAVA).

### 2.3 Microscopical study

Further examination and staining of the blood smears obtained from the Institute for Nuclear Research was carried out. Each of the blood smears was fixed and stained (Foreyt, 1989) with a commercial kit LEUCODIF 200 (Erba Lachema s.r.o., Czech Republic).

At the next stage, all blood films were examined microscopically using a Nikon model eclipse Ci-S light microscope equipped with a Jenoptik Gryphax digital camera and Gryphax image analysis system. For microscopic examination, the number of fields of view and microscope magnification was used as in Baltrūnaitė et al. (2020) and Valkiūnas (2004).

Morphometric evaluation of the detected *Trypanosoma* spp. and *Hepatozoon* spp. was performed by taking measurements using an Gryphax software. In *Trypanosoma* spp., the distance from the posterior end to the kinetoplast (PK), the posterior end to the nucleus (PN), the kinetoplast to the nucleus (KN), the width of the body with an undulating membrane (width), the width of the body without an undulating membrane (width-m), anterior end to the nucleus (AN), anterior end to the kinetoplast (AK), total pathogen body length (TL), free flagellum length (FF), nucleus length (NL), nucleus width (NW) were measured based on the studies of Borges et al. (2016), Virgilio et al. (2022) and Fonseca et al. (2023). *Hepatozoon* spp. length (BL), width (BW), nucleus length (NL), nucleus width (NW) were measured based on the study by Mansour et al. (2020).

### 2.4 DNA extraction from rodent blood films, liver and spleen tissues

DNA extraction was performed using blood films, and for available liver and spleen samples, which were stored at −20°C. In total, DNA was extracted from tissues of 32 animals. Of these only blood films were analysed for eight muroids, liver tissue alone for four individuals, and both liver and spleen samples for a further 18 individuals, while DNA samples from all tissues were available for two animals. DNA was extracted using the DNeasy Blood & Tissue kit from Qiagen (Hilden, Germany). The manufacturer's protocol was followed for total DNA extraction from liver and spleen tissues and modification of protocol was used for stained blood smears (Shavey and Morado, 2012).

### 2.5 PCR and nucleotide sequencing

Primers used and cycle conditions are shown in Table 1. The resulting PCR products were further sequenced. Sanger sequencing of positive samples was performed using internal primer sets in both directions at a commercial provider (Microsynth, Balgach, Switzerland). Sequence verification and alignments were created and adjusted in the Geneious Prime® software (version 2025.2.1; Biomatters Ltd., Auckland, New Zealand; Kearse et al., 2012). Analysis was performed using BLAST https://blast.ncbi.nlm.nih.gov/Blast.cgi and obtained results were compared. All sequences were added to the NCBI database under the following numbers PX973658, PX973671, PX973667, PX973668, PZ025234, PZ025235, PX973670, PZ020203-PZ020208 (Table 2).

**Table 1:** Primers and PCR cycle conditions used for the molecular characterization of parasites and bacteria.

Organism	Target	Primers	Sequence (5′-3′)	Length (bp)	PCR cycle conditions	References
*Trypanosoma* spp.	18S rRNA	Tryp_18S_F1 (Nest1)	GTG GAC TGC CAT GGC GTT GA	960 bp	94°C/5 min; 40 cycles: 94°C/1 min, 56°C/1 min, 72°C/1 min; 72°C/5 min	Peña-Espinoza et al. (2023)
		Tryp_18S_R1 (Nest1)	CAG CTT GGA TCT CGT CCG TTG A			
		Tryp_18S_F2 (Nest2)	CGA TGA GGC AGC GAA AAG AAA TAG AG		94°C/5 min; 40 cycles: 94°C/1 min, 56°C/1 min, 72°C/1 min; 72°C/5 min	
		Tryp_18S_R2 (Nest2)	GAC TGT AAC CTC AAA GCT TTC GCG			
*Babesia* spp*., Theileria* spp*., Hepatozoon* spp*., Cytauxzoon* spp.	18S rRNA	BTH-1F (Nest1)	CCT GAG AAA CGG CTA CCA CAT CT	700 bp	94°C/2 min; 40 cycles: 95°C/30 s, 68°C/1 min, 72°C/1 min; 72°C/10 min	Zintl et al. (2011)
		BTH-1R (Nest1)	TTG CGA CCA TAC TCC CCC CA			
		GF2F (Nest2)	GTC TTG TAA TTG GAA TGA TG		94°C/2 min; 40 cycles: 95°C/30 s, 60°C/1 min, 72°C/1 min; 72°C/10 min	
		GR2R (Nest2)	CCA AAG ACT TTG ATT TCT CT C			
*Mycoplasma* spp.	16S rRNA	HBT-F	ATA CGG CCC ATA TTC CTA CG	600 bp	94°C/2 min; 40 cycles: 95°C/1 min, 60°C/1 min, 72°C/1 min; 72°C/7 min	(Criado-Fornelio et al., 2003)
		HBT-R	TGC TCC ACC ACT TGT TCA			
*Bartonella* spp.	Citrate synthase (*gltA*)	BhCS.781p	GGG GAC CAG CTC ATG GTG G	379 bp	94°C/5 min; 40 cycles: 94°C/1 min, 54°C/1 min, 72°C/1 min; 72°C/10 min	Norman et al. (1995)
		BhCS.1137n	AAT GCA AAA AGA ACA GTA AAC A			
		GR2R (Nest2)	CCA AAG ACT TTG ATT TCT CT C			
*Ehrlichia* spp.	16S rRNA	EHR16SD-for	GGT ACC YAC AGA AGA AGT CC	345 bp	95°C/2 min; 35 cycles: 94°C/1 min, 54°C/30 s, 72°C/30 s; 72°C/5 min	Parola et al. (2000)
		EHR16SR-rev	TAG CAC TCA TCG TTT ACA GC			
*Rickettsia* spp.	17-kd antigen	Ricketts_ITS_for	GAT AGG TCG GGT GTG GAA G	400 bp	96°C/4 min; 35 cycles: 94°C/1 min, 52°C/1 min, 72°C/2 min; 72°C/3 min	Tsui et al. (2007)
		Ricketts_ITS_rev	TCG GGA TGG GAT CGT GTG

**Table 2:** List of genotypes with the animal species from whose tissues they were isolated in current research.

Genotype	Accession number	Detailed information		
		place of trapping	animal species	tissue
*Trypanosoma grosi*	PX973658	Landfill II	*A. agrarius* (n = 4)*,* *A. flavicollis* (n = 1)	liver/spleen liver/spleen
*Parabodo caudatus*-like	PX973671	Landfill II	*A. flavicollis* (n = 1)	spleen
*Hepatozoon* sp. isolate	PX973667	Landfill II	*A. flavicollis* (n = 1)	spleen
*Hepatozoon* sp. isolate	PX973668	Landfill II	*A. flavicollis* (n = 1)	spleen
Mollicutes (*Mycoplasma*-like sp.)	PZ025234	Landfill II	*A. flavicollis* (n = 1)	liver
Mollicutes (*Mycoplasma*-like sp.)	PX973670	Landfill II	*A. flavicollis* (n = 1)	spleen
Mollicutes (*Mycoplasma*-like sp.)	PZ025235	Landfill II	*A. agrarius* (n = 2)	liver/spleen
Uncultured *Bartonella* sp.	PZ020203	Landfill II	*A. sylvaticus* (n = 1)	spleen
Uncultured *Bartonella* sp.	PZ020204	Landfill II	*A. sylvaticus* (n = 2), *A. flavicollis* (n = 2)	liver/spleen liver/spleen
Uncultured *Bartonella* sp.	PZ020205	Landfill II	*A. agrarius* (n = 1), *A. flavicollis* (n = 1)	blood film liver
Uncultured *Bartonella* sp.	PZ020206	Chernechyi Lis (Monk's Forest)	*M. glareolus* (n = 4), *A. agrarius* (n = 1), *A. flavicollis* (n = 2), ND (n = 1)	blood film blood film blood film blood film
		Landfill II	*A. flavicollis* (n = 1)	blood film
		Landfill III	*A. agrarius* (n = 1), *A. flavicollis* (n = 3), *A. sylvaticus* (n = 1), ND (n = 2)	liver/spleen liver/spleen liver liver/spleen
Uncultured *Bartonella* sp.	PZ020207	Landfill III	*M. glareolus* (n = 1)	liver/spleen
Uncultured *Bartonella* sp.	PZ020208	Chernechyi Lis (Monk's Forest)	*M. glareolus* (n = 1), *A. agrarius* (n = 1), ND (n = 1)	blood film blood film blood film
		Landfill II	*A. flavicollis* (n = 1)	blood film
		Landfill III	*A. flavicollis* (n = 2)	liver/spleen

Species identification of 19 muroid rodents not morphologically identified was done by DNA barcoding with the help of mitochondrial gene for cytochrome *b* (Galan et al., 2012). For this reason, DNA extracted from liver samples and blood films was used.

Phylogenetic analysis was performed using Geneious Prime® 2025.2.1 software programme. For comparison with the genotypes obtained in our study, additional genotypes were retrieved from GenBank using the BLAST function. After the selected sequences were aligned using the MAFFT algorithm and the poorly aligned terminal regions were trimmed manually, a maximum likelihood analysis was conducted using IQ-TREE version 2.3.6. Automatic substitution model selection via ModelFinder was employed. Node support was assessed using 1000 ultrafast bootstrap replicates with nearest neighbour interchange optimisation.

### 2.6 Measurement of radionuclide levels in the air, soil and bodies of rodents

Radionuclide levels in air (PEDγ, β-particle flux density), in soil (^137^Cs, ^90^Sr, ^241^Am) and ^137^Cs in animal organisms were measured in accordance with the previously described methods (Burdo et al., 2020).

### 2.7 Statistical analysis

Mean ± SD, minimum, and maximum values of parasites based on the results of microscopic examination were calculated in Excel (Microsoft, USA, Version 2408 Build 16.0.17932.20286) and rounded to two decimals.

The confidence intervals (95% CI) for proportions were calculated using OpenEpi (Dean et al., 2018), as well as for the generation of graphical data representation.

Python 3.9.6 with relevant libraries Pandas 1.5.3, SciPy 1.11.1 and PyCharm 2024.3.6 (Professional Edition) was used for the statistical analysis of the obtained data. An independent t-tests was applied to compare groups of animals by sex, while all other groups of animals were compared using one-way ANOVA. For comparison of the prevalence of parasites and bacteria between the various trapping sites and the control site, was used Fisher's exact test.

## 3 Results

### 3.1 Microscopy

**Fig. 2:**
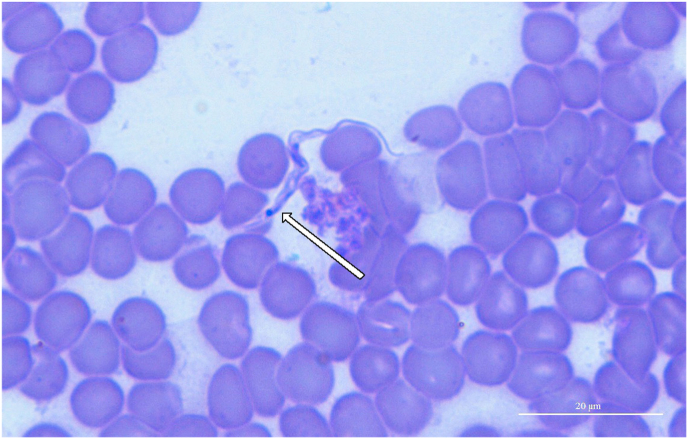
Trypomastigote of *T. grosi* in blood smear of *A*. *agrarius*.

**Fig. 3:**
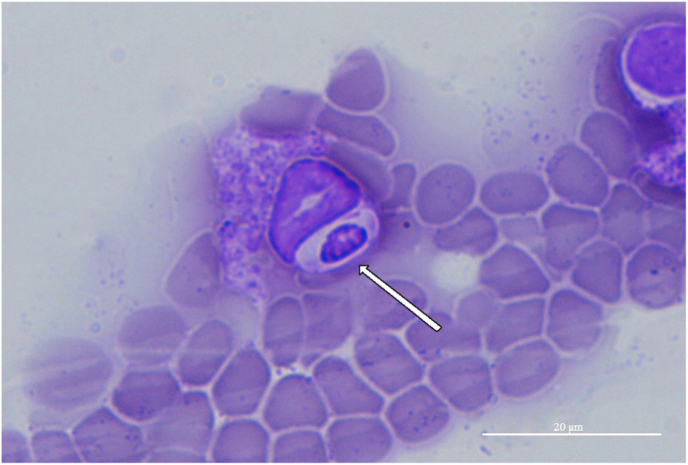
*Hepatozoon* sp. gametocyte in blood smear of *A*. *flavicollis*.

According to the results of microscopy, *Trypanosoma* spp. (Fig. 2) were found in 9 (7.8%, 95% CI 3.9-13.8) muroid rodents, *Hepatozoon* spp. (Fig. 3) in 14 (12.1%, 95% CI 7.0-19.0) animals and agents of bacterial origin in 66 (56.9%, 95% CI 47.8-65.7) animals. Microscopic measurements were performed for the identified *Trypanosoma* spp. and *Hepatozoon* spp. (Table 3, Table 4).

**Table 3:** Measurements of the microscopically and/or molecularly identified *Trypanosoma* spp.

Muroid rodent species	n *Trypanosoma* spp. in an individual animal blood film	Parameter	PK	PN	KN	width	width-m	AN	AK	tL	FF	nucleus length	nucleus width
*Apodemus agrarius*	13 (*T. grosi,* AN PX973658)	Mean, μm	4.71 ± 0.82	11.35 ± 1.15	5.89 ± 1.69	2.04 ± 0.42	1.09 ± 0.32	10.08 ± 1.46	19.19 ± 2.17	32.59 ± 1.95	8.23 ± 1.37	2.92 ± 0.38	0.99 ± 0.18
		Min., μm	2.88	8.05	2.1	1.36	0.63	6.95	13.62	27.52	5.96	2.16	0.73
		Max., μm	5.8	12.77	7.75	2.81	1.77	11.8	21.76	34.76	9.74	3.59	1.4
	4 (*T. grosi,* AN PX973658)	Mean, μm	3.3 ± 0.58	11.8 ± 0.56	8.31 ± 0.68	2.29 ± 0.4	1.37 ± 0.55	8.07 ± 1.23	18.87 ± 1.66	31.17 ± 1.26	8.05 ± 1.27	3.24 ± 0.62	1.14 ± 0.26
		Min., μm	2.68	11.47	7.31	1.74	0.85	6.88	17.12	29.85	6.77	2.33	0.84
		Max., μm	3.9	12.64	8.84	2.69	2.08	9.49	20.33	32.64	9.79	3.64	1.46
	6 (*T. grosi,* AN PX973658)	Mean, μm	3.71 ± 0.78	11.18 ± 1.28	7.44 ± 1.19	2.12 ± 0.36	1.18 ± 0.51	8.15 ± 2.85	18.1 ± 3.45	28.92 ± 3.96	6.72 ± 1.52	2.88 ± 0.38	1.1 ± 0.29
		Min., μm	3.01	9.25	5.3	1.74	0.74	4.9	13.5	24.43	4.84	2.32	0.72
		Max., μm	4.79	12.54	8.79	2.72	2.12	11.25	21.96	33.41	8.3	3.31	1.56
Mean, μm			4,2 ± 0,94	11,39 ± 1,07	6,72 ± 1,68	2,11 ± 0,39	1,16 ± 0,4	9,23 ± 2,01	18,85 ± 2,37	31,38 ± 2,83	7,8 ± 1,45	2,97 ± 0,42	1,04 ± 0,22
*Apodemus flavicollis*	1	μm	2.71	9.21	6.08	1.67	0.69	4.98	12.83	19.75	3.78	1.77	0.73
	11	Mean, μm	3.72 ± 0.35	11.73 ± 0.51	7.21 ± 0.41	1.84 ± 0.22	1.09 ± 0.2	9.94 ± 1.38	19.74 ± 1.18	31.59 ± 1	7.32 ± 1.02	2.6 ± 0.3	0.83 ± 0.1
		Min., μm	3.33	11.04	6.69	1.58	0.76	7.28	17.26	30.04	5.97	2.05	0.72
		Max., μm	4.65	12.66	7.85	2.22	1.38	11.55	21.62	33.16	9.04	3	0.98
*Apodemus* sp.	71	Mean, μm	4.11 ± 0.9	12.48 ± 1.31	7.71 ± 1.05	2.22 ± 0.35	1.18 ± 0.28	10.47 ± 2.02	21.1 ± 2.09	33.31 ± 2.33	7.42 ± 1.5	2.95 ± 0.57	1.01 ± 0.22
		Min., μm	1.97	8.7	4.72	1.44	0.59	5.89	15.67	25.54	3.79	1.66	0.61
		Max., μm	6.34	15.63	10.97	3.26	1.9	14.65	25.14	37.24	10.16	4.38	1.64
*Myodes glareolus*	20	Mean, μm	3.75 ± 0.64	9.74 ± 0.85	5.25 ± 0.95	1.82 ± 0.29	0.97 ± 0.15	8.55 ± 1.28	17.01 ± 1.53	28.86 ± 1.99	7.39 ± 1.15	3.18 ± 0.8	0.89 ± 0.2
		Min., μm	2.36	8.09	4.25	1.25	0.66	5.78	14.54	25.23	5.38	1.92	0.61
		Max., μm	4.85	11.63	7.81	2.3	1.25	10.93	20.43	32.54	9.85	4.53	1.27

Explanations: PK – posterior end to the kinetoplast, PN – posterior end to the nucleus, KN – kinetoplast to the nucleus, width – width of the body with an undulating membrane, width-m –

width of the body without an undulating membrane, AN – anterior end to the nucleus, AK – anterior end to the kinetoplast, tL – total pathogen body length, FF - free flagellum length, NL – nucleus length, NW – nucleus width.

**Table 4:** Measurements of the microscopically identified *Hepatozoon* spp.

Muroid rodent species	n *Hepatozoon* spp. in an individual animal blood film	Parameter	BL	BW	NL	NW
*Apodemus flavicollis*	19	Mean, μm	10.77 ± 0.47	4.49 ± 0.37	5.12 ± 0.7	2.72 ± 0.49
		Min., μm	10.05	3.79	4.12	1.65
		Max., μm	11.64	5.27	6.9	3.74
*Apodemus flavicollis*	2	Mean, μm	10.79 ± 0.47	4.5 ± 0.01	4.62 ± 0.97	2.56 ± 0.28
		Min., μm	10.46	4.5	3.93	2.37
		Max., μm	11.12	4.51	5.31	2.76
*Apodemus flavicollis*	10	Mean, μm	10.54 ± 0.24	4.83 ± 0.2	5.05 ± 0.82	3.53 ± 0.39
		Min., μm	10.13	4.61	3.5	2.92
		Max., μm	10.91	5.21	6.28	4.19
Mean		μm	10,7 ± 0,41	4,6 ± 0,34	5,06 ± 0,72	2,97 ± 0,58
*Myodes glareolus*	1	μm	10.16	3.99	4.55	2.26
*Myodes glareolus*	5	Mean, μm	9.69 ± 0.51	4.09 ± 0.16	5.13 ± 0.81	3.29 ± 0.59
		Min., μm	9.14	3.83	4.24	2.8
		Max., μm	10.32	4.23	5.82	3.94
*Myodes glareolus*	26	Mean, μm	9.22 ± 0.72	4.07 ± 0.5	4.65 ± 0.85	2.47 ± 0.41
		Min., μm	7.76	2.89	2.36	1.71
		Max., μm	10.5	4.93	6.45	3.31
*Myodes glareolus*	73	Mean, μm	10.48 ± 0.72	3.97 ± 0.36	4.98 ± 0.55	2.76 ± 0.35
		Min., μm	9.12	3.32	3.71	1.79
		Max., μm	11.94	4.74	7.09	3.84
*Myodes glareolus*	62	Mean, μm	10.25 ± 1.07	4.09 ± 0.37	5.07 ± 0.76	2.88 ± 0.35
		Min., μm	8.32	3.15	3.2	1.97
		Max., μm	13.74	4.9	6.84	3.61

Explanations: BL – length of gametocyte, BW – width of gametocyte, NL – nucleus length, NW – nucleus width.

### 3.2 Results of PCR and further sequencing

#### 3.2.1 Trypanosoma grosi

According to the results of PCR, 10 positive animals (31.25%, 95% CI 17.09-48.67) were identified among 32 animals tested for *Trypanosoma*.

**Fig. 4:**
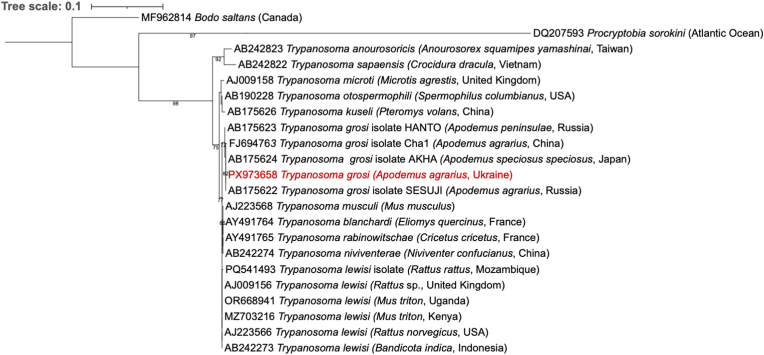
Maximum likelihood phylogenetic tree of *T. grosi* genotypes based on the 18S rRNA locus. The outgroup species used was *Bodo saltans*. The genotypes detected in our research are marked in red. Bootstrap values ≥ 70 are shown at nodes. Scale bar indicates substitutions per site.

Four animals, one *A*. *agrarius* and three *M*. *glareolus* tested positive by PCR based on blood films, but no sequencing data were obtained. The other genotypes obtained from five liver and spleen samples of four *A*. *agrarius* and one *A*. *flavicollis* showed 100% sequence identity to *Trypanosoma grosi* Laveran and Pettit, 1909 isolates AKHA with AN AB175624 from *Apodemus speciosus speciosus* (Temminck, 1844) (Takko, Aomori, Japan) and SESUJI with AN AB175622 from *A*. *agrarius* (vladivostok, russia) (Sato et al., 2005). Our sequence under AN PX973658 showed 99.89% (one sequence), 99.78% (one sequence), and 99.56% identity (10 sequences) to reference sequences with AB175623 (isolate HANTO, *Apodemus peninsulae* (Thomas, 1907), vladivostok, Russia), FJ694763 (*A*. *agrarius*, China), OR452746 (*Hylomyscus denniae* (Thomas, 1906), Uganda), MZ703216 (*Mus triton* Thomas, 1909; Kenya), AJ009156 (isolate Molteno B3), MZ703221 (*Lemniscomys striatus* (Linnaeus, 1758), Kenya), AJ223566 (*Rattus norvegicus* (Berkenhout, 1769), USA), OR668941 (*M*. *triton*, Uganda), MZ703217 (*Stenocephalemys albipes* (Rüppell, 1842), Ethiopia), OR668943 (*Lophuromys stanleyi* (Verheyen, Hulselmans, Dierckx, Mulungu, Leirs, Corti & Verheyen, 2007) Uganda), OR668946 (*Crocidura* sp., Uganda) and AB242273 (*Bandicota indica* (Bechstein, 1800), Indonesia) (Sato et al., 2005; Guan et al., 2011; Babyesiza et al., 2024; Votýpka et al., 2022; Stevens et al., 1998; Haag et al., 1998; Mafie et al., 2019). According to the phylogenetic analysis, the genotype obtained in our study shows 87% bootstrap support and clusters together with other genotypes of *T. grosi*, as shown in Fig. 4.

Among the five animals with microscopically identified *Trypanosoma* sp., parasite DNA presence was confirmed by PCR. Subsequent sequencing was successful for tissues from only four animals, with blood films from three of these being available for morphometric measurements (Table 3).

#### 3.2.2 *Hepatozoon* spp.

**Fig. 5:**
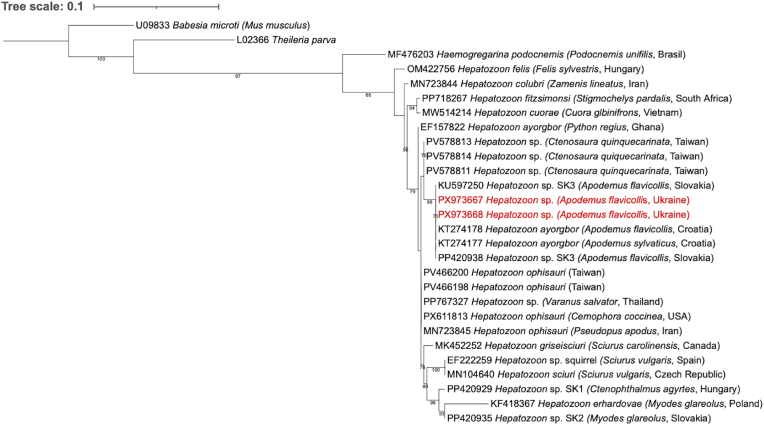
Maximum likelihood phylogenetic tree based on 18S rRNA locus showing the position of *Hepatozoon* sp. detected in the present study (red) among selected *Hepatozoon* spp. and related apicomplexan parasites. *Babesia microti* and *Theileria parva* were included as outgroup taxa. Bootstrap values ≥ 70 are shown at nodes. Scale bar indicates substitutions per site.

Altogether, tissues from 32 rodents were examined by PCR to detect the presence of *Hepatozoon* spp./*Babesia* spp./*Theileria* spp. DNA. Among the 10 (31.25%, 95% CI 17.09-48.67) positive animals, samples of two of them were successfully genotyped and deposited in NCBI under AN PX973667 and PX973668 (Table 2) with nucleotide identity 99.06% and 98.94% to *Hepatozoon ayorgbor* Sloboda et al. (2007) with AN EF157822 (Sloboda et al., 2007), 98.94% and 98.82% to *Hepatozoon* sp. (MZ412878; Jameie et al., 2022) and *Hepatozoon ophisauri* (Tartakovskii, 1913) (MN723845; Zechmeisterová et al., 2021), 98.70% and 98.58% to *Hepatozoon* sp. with AN MT919387, MT919388, FJ719818 and FJ719817 (Hrazdilová et al., 2021; Merino et al., 2009) and 98.59% and 98.47% with AN FJ719819, PQ807554 and PQ807539 (Merino et al., 2009; Freitas et al., 2025). Based on the results of the phylogenetic analysis, both *Hepatozoon* genotypes obtained in our study form a distinct clade with previously described genotypes SK3 (KU597250 and PP420938) showing strong bootstrap support (96%) (Fig. 5).

All blood films and liver samples were negative, while positive results were obtained only from animal spleen samples. *Hepatozoon* genotypes identified by sequencing were obtained from *A*. *flavicollis* captured at landfill II. Moreover, in both samples from which sequences were obtained, no *Hepatozoon* were detected microscopically. Among all samples tested by PCR and subsequently sequenced, only one animal unidentified to species female of the genus *Apodemus*, weighing 13.54 g, trapped at landfill III in September was found to have a genotype corresponding to *Babesia canis* (Piana and Galli-Valerio, 1895). Further examination of this animal's blood film using the PCR yielded a negative result.

#### 3.2.3 Mollicutes (*Mycoplasma*-like spp.)

**Fig. 6:**
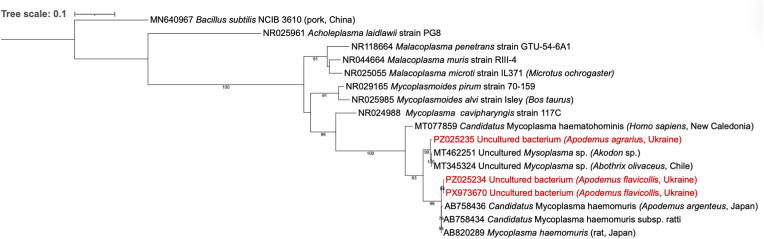
Maximum likelihood phylogenetic tree based on 16S rRNA locus of Mollicutes (*Mycoplasma*-like spp.). The outgroup species used was *Bacillus subtilis*. The genotypes detected in our research are marked in red. Bootstrap values ≥ 70 are shown at nodes. Scale bar indicates substitutions per site.

We also identified five positive samples (15.6%, 95% CI 6.0-31.3) when analysing the 16S rRNA *Mycoplasma* gene (600 bp) among the 32 samples subjected to a PCR with primers HBT-F and HBT-R (Table 1). For one sample, DNA was successfully isolated from the spleen, and for two samples from the liver, while in the other two samples both the spleen and the liver tested positive. Further sequencing of all positive samples revealed three different genotypes (Table 2, Fig. 6). One genotype with 557 base pairs was found among *A*. *agrarius*, and other two in *A*. *flavicollis*. The first genotype (PZ025234) detected was most similar (99.46% of identity) to a partial 16S rRNA gene sequence of an Uncultured *Mycoplasma* clone mink766 with AN MT462251, which was isolated from American minks (*Neovision vison* (Schreber, 1777)) (Sepúlveda-García et al., 2021). The subsequent 13 genotypes (KT215623, KT215621, MT345324, KT215630, KT215624, MT345318, KT215629, KT215622, PP109115, PP109116, KT215627, PP109117, KT215628) from GenBank showed 98.92%-99.28% identity with the one we obtained (Gonçalves et al., 2015; Alabí et al., 2020; Machado et al., 2024). Second genotype (PX973670) was 99.41% similar to 12 previously deposited genotypes (AB758439 Fukushima, Japan, black rat; U82963, Japan, wild mice; ON733033, Gauteng Province, South Africa, *Rattus tanezumi* (Temminck, 1844); MN423261, Brazil, *Rattus rattus* (Linnaeus, 1758); AB758436, Fukushima, Japan, field mouse; KM258432, MN423265, Brazil, *Polyplax spinulosa* (Burmeister, 1839) (louse); AB918692 ‘Candidatus *Mycoplasma haemomuris* subsp. *musculi*’ (basonym *M*. *haemomuris* (Mayer, 1921)), small field mouse, Japan; AB758435, ‘Candidatus *Mycoplasma haemomuris* subsp. *ratti*’ (basonym *M*. *haemomuris*), black rat, Okinawa, Ikema Island, Japan; KT215635, Uncultured *Mycoplasma* sp., wild rodent, Brazil; AB758440, Candidatus *M*. *haemomuris* subsp. *musculi*, *Apodemus argenteus* (Temminck, 1844), Japan; AB758434, Candidatus *M*. *haemomuris* subsp. *ratti*, black rat, Okinawa, Ikema Island, Japan), which mainly belonged to Candidatus *M*. *haemomuris*, formely *Haemobartonella muris* (Mayer, 1921)*,* uncultured *Mycoplasma* sp. (Rikihisa et al., 1997; Retief et al., 2022; Gonçalves et al., 2015, 2020; Conrado et al., 2015; Harasawa et al., 2015) and were isolated from black rat, wild mice etc., whereas the third genotype (PZ025235) showed 99.54% sequence identity to the seven previously deposited genotypes with AN AB758439, ON733033, MN423261, KM258432, AB758435, KT215635, AB758434 similar to the second genotype, it also clustered with five newly obtained sequences (OP271911: Uncultured *Mycoplasma* sp., *R*. *rattus*, Northeastern Brazil; OP271912: Uncultured *Mycoplasma* sp., *R*. *rattus*, Northeastern Brazil; OP954342: Azara's agoutis (*Dasyprocta azarae* Lichtenstein, 1823), Southern Brazil; AB820289: *H*. *muris*, rat, Japan; MK959182: Uncultured *Mycoplasma* sp., rat, Malaysia) (Torres-Santos et al., 2024; Elshafie et al., 2024; Low et al., 2020). Phylogenetic analysis showed that the two genotypes obtained in our study (PX973670 and PZ025234) belonged to a sister clade that also contained the third genotype (PZ025235) (Fig. 6). These results are supported by strong bootstrap support (94-99%).

#### 3.2.4 *Bartonella* spp.

**Fig. 7:**
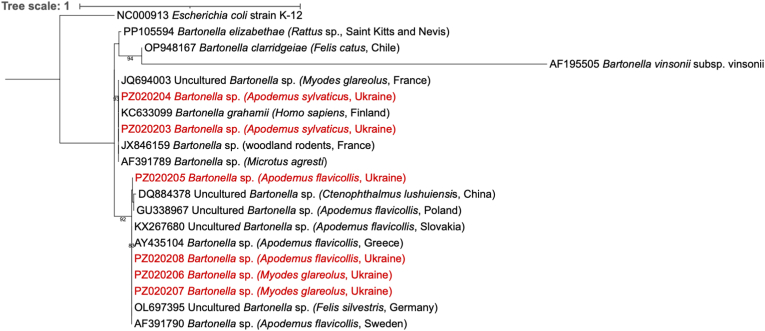
Maximum likelihood phylogenetic tree based on the gltA locus of *Bartonella* spp. The outgroup specie used was *Escherichia coli*. The genotypes detected in our research are marked in red. Bootstrap values ≥ 70 are shown at nodes. Scale bar indicates substitutions per site.

The presence of *Bartonella* spp. was identified in 26 (81.3%, 95% CI 64.0-92.0) of the muroid rodents that were analysed. Six different genotypes (AN PZ020203-PZ020208, Table 2, Fig. 7) were present within mentioned *Bartonella* isolates. One of the genotypes (AN PZ020206) was found in 12 animals at once, including species such as *M*. *glareolus*, *A. agrarius*, *A*. *flavicollis* and *A*. *sylvaticus*. Based on sequence comparison with entries in the NCBI database, we found a 99.11% similarity with 99% of query coverage to an uncultured *Bartonella* sp. with AN KX267680 (*A*. *flavicollis*, Slovakia), 98.82% similarity (100% and 99% of coverage) with AN CP083444 (*Bartonella taylorii* Birtles et al., 1995 strain IBS296), DQ155393 (*A*. *flavicollis*, Slovenia), CP083693 (*B*. *taylorii*, *A*. *sylvaticus*, United Kingdom), AY435108 (*A*. *flavicollis*, Greece), AY435104 (*A*. *flavicollis*, Greece), JQ694004 (*M*. *glareolus*, France) and KX267683 (*A*. *flavicollis*, Slovakia) (Kraljik et al., 2016; Siewert et al., 2022; Knap et al., 2007; Buffet et al., 2012). The next most common was the genotype under AN PZ020208, which we found in 6 animals. It is identical to the mentioned above genotypes KX267680 (100%), AY35108, AY35104, JQ694004, CP083444, DQ155393, CP083693, KX267683 (99.70% similarity). In 4 animals, we found a genotype PZ020204 with zoonotic importance. Its identity with *Bartonella grahamii* Birtles et al., 1995 genotype as4aup deposited in GenBank under AN CP001562 was 100% (Berglund et al., 2009). The same level of identity was also observed for the genotype with accession number AN JQ694003 (Buffet et al., 2012). We identified the next three genotypes in each individual animal. Of these genotypes, genotype under AN PZ020203 isolated from *A*. *sylvaticus* was 99.41% similar to *B*. *grahamii* under AN AB426654 (*Myodes gapperi* (Vigors, 1830), Canada (Inoue et al., 2009)), KC633099 (immunocompromised patient, Finland (Oksi et al., 2013)). Genotype PZ020205 from *A*. *flavicollis* and *A. agrarius* showed 99.15% identity to deposited genotypes GU338967 (*A*. *flavicollis*, Poland), KX267680 (*A*. *flavicollis*, Slovakia), AF391790 (Sweden), AY435113 and AY435111 (*A*. *flavicollis*, Greece), KF546311 (flea *Ctenophtalmus agyrtes* (Heller, 1896), Lithuania), JQ694022 (*M*. *glareolus*, France), GU338965 (*M*. *glareolus*, Poland), OQ305232 (*Apodemus witherbyi* (Thomas, 1902), Turkey) and others (Paziewska et al., 2011; Kraljik et al., 2016; Holmberg et al., 2003; Buffet et al., 2012; Çelebi et al., 2015). The last *Bartonella* sp. genotype (PZ020207) from *M*. *glareolus* was 99.70% similar to JQ694012 (*M*. *glareolus*, France) and PV170757 (Czechia) and 99.41% similar to AF391790 (Sweden) and KX267679 (*M*. *glareolus*, Slovakia) (Buffet et al., 2012; Holmberg et al., 2003; Kraljik et al., 2016). According to the results of the phylogenetic analysis, the two *Bartonella* genotypes (PZ020204 and PZ020203) obtained in our study belong to the clade with *Bartonella grahamii* genotype (strong bootstrap support, 93%), whereas the other four genotypes belong to another clade that is sister to this one (Fig. 7).

#### 3.2.5 *Rickettsia* spp., *Ehrlichia* spp. and *Toxoplasma gondii*

PCR was used to detect *Rickettsia* spp. in one (3.1%, 95% CI 0.2-14.5) out of 32 animals, but sequencing could not confirm this. *Ehrlichia* spp. and *Toxoplasma gondii* Nicolle and Manceaux, 1908, were not detected in any of the studied animals.

### 3.3 Parasites and bacteria according to the species of animals

According to the research findings, *Trypanosoma* spp. (4.1%, microscopy) and *T. grosi* (9.1%, PCR, subsequent sequencing), *Hepatozoon* spp. (6.1%, microscopy), Mollicutes (*Mycoplasma*-like spp.) (9.1% by PCR, subsequent sequencing), and *Bartonella* spp. (81.8% by PCR) were detected in *A*. *flavicollis*. At the same time, *Trypanosoma* spp*.* (15.8% according to the microscopy) and *T. grosi* (50.0% with the help of PCR), Mollicutes bacteria (50% detected by PCR), and *Bartonella* spp. (37.5% detected by PCR) were found in mice *A*. *agrarius*, whereas in *M*. *glareolus*, *Trypanosoma* spp. (9.7% according to the microscopy), *Hepatozoon* spp. (35.5% according to the microscopy), and *Bartonella* spp. (100% according to PCR) were identified. In *A*. *sylvaticus*, only *Bartonella* spp. (80% according to PCR) were detected. Statistically significant association was found between muroid species and the prevalence rate according to the microscopy of *Hepatozoon* spp. (Table 5, Table 6, Table 7, 10, Supplementary Material) (*p* < 0.001), whereas PCR followed by genotyping through sequencing revealed an association with the prevalence of *T*. *grosi* (*p* = 0.009) and *Bartonella* spp. (*p* < 0.001).

**Table 5:** The prevalence of parasites and bacteria in *Myodes glareolus* (n = 31) using microscopy and PCR, with subsequent confirmation by sequencing.

Parasite/bacteria		Total	Trapping in			Sex		Weight		
			July	September	October	female	male	up to 15 g	15-21 g	21-27 g
Microscopy										
*Trypanosoma* spp.	n positive animals/n tested	3/31	2/11	0/8	1/12	3/14	0/17	1/2	0/17	2/11
	% (95% CI)	9.7 (2.5-24.1)	18.2 (3.2-48.3)	0	8.3 (0.4-34.8)	21.4 (5.8-48.0)	0	50 (2.5-97.5)	0	18.2 (3.2-48.3)
	*p* value	0.763	0.434			0.046		0.088		
*Hepatozoon* spp.	n positive animals/n tested	11/31	3/11	2/8	6/12	4/14	7/17	1/2	4/17	6/11
	% (95% CI)	35.5 (20.3-53.3)	27.3 (7.5-57.8)	25.0 (4.4-61.2)	50.0 (23.4-76.6)	28.6 (9.8-55.6)	41.2 (20.1-65.0)	50 (2.5-97.5)	23.5 (8.0-47.5)	54.5 (25.9-81.0)
	*p* value	0.001	0.430			0.482		0.343		
PCR										
*Trypanosoma* sp./*T. grosi; Hepatozoon* spp.; *Mycoplasma*-like spp.	n positive animals/n tested	0/5	0/4	0/1	0/0	0/4	0/1	0/0	0/1	0/4
*Bartonella* spp.	n positive animals/n tested	5/5	4//4	1/1	0/0	4/4	1/1	0/0	1/1	4/4
	% (95% CI)	100.0 (54.9-100.0)	100.0 (47.3-100.0)	100.0 (5.0-100.0)	0	100.0 (47.3-100.0)	100.0 (5.0-100.0)	0	100.0 (5.0-100.0)	100.0 (47.3-100.0)
	*p* value	<0.001	nd			0.453		nd

**Table 6:** The prevalence of parasites and bacteria in *Apodemus agrarius* (n = 19) using microscopy and PCR, with subsequent confirmation by sequencing.

Parasite/bacteria		Total	Trapping in			Sex		Weight		
			July	September	October	female	male	up to 15 g	15-21 g	21-27 g
Microscopy										
*Trypanosoma* spp.	n positive animals/n tested	3/19	0/4	3/14	0/1	0/8	3/11	2/10	1/3	0/6
	% (95% CI)	15.8 (4.2-37.2)	0	21.4 (5.8-48.0)	0	0	27.3 (7.5-57.8)	20.0 (3.5-52.0)	33.3 (1.7-86.8)	0
	*p* value	0.763	0.574			0.120		0.420		
*Hepatozoon* spp.	n positive animals/n tested	0/19	0/4	0/14	0/1	0/8	0/11	0/10	0/3	0/6
PCR										
*Trypanosoma* sp./*T. grosi*	n positive animals/n tested	4/8	0/1	4/7	0/0	1/2	3/6	2/4	1/2	1/2
	% (95% CI)	50.0 (18.4-81.6)	0	57.1 (21.6-87.7)	0	50.0 (2.5-97.5)	50.0 (14.7-85.3)	50.0 (9.4-90.6)	50.0 (2.5-97.5)	50.0 (2.5-97.5)
	*p* value	0.009	nd			1.0		1.0		
*Hepatozoon* spp.	n positive animals/n tested	0/8	0/1	0/7	0/0	0/2	0/6	0/4	0/2	0/2
*Mycoplasma*-like spp.	n positive animals/n tested	2/8	0/1	2/7	0/0	0/2	2/6	2/4	0/2	0/2
	% (95% CI)	25.0 (4.4-61.2)	0	28.6 (5.1-67.0)	0	0	33.3 (6.0-73.8)	50.0 (9.4-90.6)	0	0
	*p* value	0.620	nd			0.420		0.363		
*Bartonella* spp.	n positive animals/n tested	3/8	1/1	2/7	0/0	0/2	3/6	1/4	1/2	1/2
	% (95% CI)	37.5 (10.6-72.2)	100.0 (5.0-100.0)	28.6 (5.1-67.0)	0	0	50.0 (14.7-81.3)	25.0 (1.3-75.8)	50.0 (2.5-97.5)	50.0 (2.5-97.5)
	*p* value	<0.001	nd			0.267		0.842

**Table 7:** The prevalence of parasites and bacteria in *Apodemus flavicollis* (n = 49) using microscopy and PCR, with subsequent confirmation by sequencing.

Parasite/bacteria		Total	Trapping in			Sex			Weight			
			July	September	October	female	male	nd	up to 15 g	15-21 g	21-27 g	over 27 g
Microscopy												
*Trypanosoma* spp.	n positive animals/n tested	2/49	0/8	1/20	1/21	0/30	1/18	1/1	1/8	0/12	1/12	0/17
	% (95% CI)	4.1 (0.7-12.8)	0	5.0 (0.2-22.3)	4.8 (0.2-21.3)	0	5.6 (0.3-24.5)	100.0 (5.0-100.0)	12.5 (0.6-48.0)	0	8.3 (0.4-34.8)	0
	*p* value	0.763	0.825			<0.001			0.375			
*Hepatozoon* spp.	n positive animals/n tested	3/49	1/8	2/20	0/21	2/30	1/18	0/1	0/8	2/12	1/12	0/17
	% (95% CI)	6.1 (1.6-15.8)	12.5 (0.6-48.0)	10.0 (1.7-29.3)	0	6.7 (1.1-20.3)	5.6 (0.3-24.5)	0	0	16.7 (2.9-49.1)	8.3 (0.4-34.8)	0
	*p* value	0.001	0.306			0.958			0.269			
PCR												
*Trypanosoma* sp./*T. grosi*	n positive animals/n tested	1/11	0/2	1/9	0/0	0/8	0/2	1/1	0/2	1/5	0/3	0/1
	% (95% CI)	9.1 (0.5-37.3)	0	11.1 (0.6-48.0)	0	0	0	100 (5.0-100.0)	0	20.0 (1.1-70.1)	0	0
	*p* value	0.009	0.169			nd			0.812			
*Hepatozoon* spp.	n positive animals/n tested	2/11	0/2	2/9	0/0	1/8	1/2	0/1	1/2	0/5	0/3	1/1
	% (95% CI)	18.2 (3.2-48.3)	0	22.2 (3.9-56.2)	0	12.5 (0.6-48.0)	50.0 (2.5-97.5)	0	50.0 (2.5-97.5)	0	0	100.0 (5.0-100.0)
	*p* value	0.371	0.169			0.498			0.032			
*Mycoplasma*-like spp.	n positive animals/n tested	2/11	0/2	2/9	0/0	0/8	1/2	1/1	0/2	1/5	0/3	0/1
	% (95% CI)	18.2 (3.2-48.3)	0	22.2 (3.9-56.2)	0	0	50.0 (2.5-97.5)	100 (5.0-100.0)	0	20.0 (1.1-70.1)	0	0
	*p* value	0.620	0.347			0.009			0.149			
*Bartonella* spp.	n positive animals/n tested	10/11	2/2	8/9	0/0	7/8	2/2	0/1	1/2	4/5	3/3	1/1
	% (95% CI)	90.9 (62.7-99.6)	100.0 (22.4-100.0)	88.9 (56.1-99.4)	0	87.5 (52.0-99.4)	100.0 (22.4-100.0)	0	50.0 (2.5-97.5)	80.0 (29.9-98.9)	100.0 (36.8-100.0)	100.0 (5.0-100.0
	*p* value	<0.001	0.347			nd			0.812			

The prevalence rates of parasites and bacteria varied among animals of different sexes and age groups. Specifically, females of *M*. *glareolus* showed a higher level of *Trypanosoma* spp. infection compared to males, while in *A*. *flavicollis*, the infection rates were nearly the same in both sexes (Table 5, Table 6, Table 7, Supplementary Material). They were statistically significant for these parameters.

### 3.4 Co-infections

In twelve (37.5%, 95% СІ 22.2-55.0) muroid rodents, concurrent infection with several pathogens was detected (Table 5, Table 6, Table 7). Thus, *T. grosi* and *Bartonella* spp. were detected in three *M*. *glareolus*. *Trypanosoma* spp., *Hepatozoon* spp. and *Mycoplasma* spp. were detected in two *A*. *agrarius*, with *Bartonella* spp. additionally to the above-mentioned pathogens detected in one animal. In four *A*. *flavicollis* individuals, distinct co-infection patterns were observed: *Hepatozoon* spp. and *Bartonella* spp. (n = 1); *Hepatozoon* spp., *Bartonella* spp., and Mollicutes (*Mycoplasma*-like spp.) (n = 1); *Hepatozoon* spp., *Bartonella* spp. and *Trypanosoma* spp. (n = 1); *Hepatozoon* spp., *Bartonella* spp., *Trypanosoma* spp., and Mollicutes (*Mycoplasma*-like bacteria) (n = 1). *Hepatozoon* spp. and Mollicutes were detected in one of the mice, which was not identified to species.

### 3.5 Parasites and bacteria according to the locations where animals with varying levels of radioactive contamination were caught

**Fig. 8:**
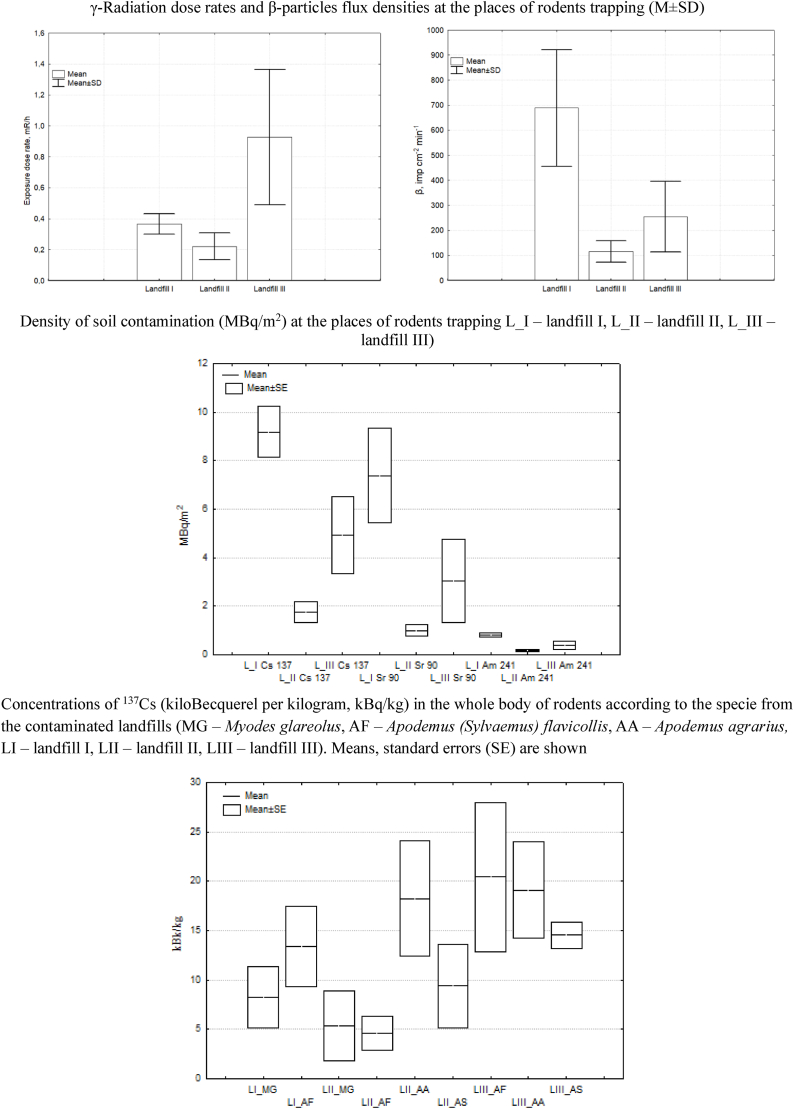
Radioactive contamination on the territo the territory of the trapping sites (landfills I, II and III), including soil contamination and the ^137^Cs concentrations within rodent organism according to the species of muroids.

Four out of five rodent-trapping places were located within the ChEZ, namely the Red Forest and all three landfills. At the same time, 42 rodents were caught in the Chernechyi Lis, more than 100 km from the Exclusion Zone (Fig. 1), in order to compare the prevalence of parasites and bacteria in animals. Table 8, Table 9 presents the data on the prevalence of pathogens in relation to the place where the animals were trapped. These data were statistically significant based on the microscopic findings and the number of animals positive for *Hepatozoon* spp. (*p* < 0.001). In contrast, for the PCR data followed by sequencing, statistical significance was demonstrated for *Trypanosoma* spp. (*p* = 0.005), Mollicutes (*p* = 0.019) and *Bartonella* spp. (*p* < 0.001) (Table 10). Radioactive contamination of the trapping sites was assessed, including soil measurements, as well as determination of ^137^Cs levels in the bodies of animals. The results of the measurements are visualised in Fig. 8.

**Table 8:** The prevalence of parasites and bacteria in muroid rodents using microscopy depending on the place of capture (*Mg* – *Myodes glareolus*, *Af* – *Apodemus flavicollis*, *Aa* – *Apodemus agrarius*, *As* – *Apodemus sylvaticus*, *Au* – *Apodemus uralensis*, *Mm* – *Mus musculus*, ND – not identified).

Place of rodent trapping	n animals	*Trypanosoma* spp.			*Hepatozoon* spp.		
		n positive animals	in % (95% CI)	*p* value	n positive animals	in % (95% CI)	*p* value
Rudyi Lis (Red Forest) (trapping in October)	20 (19 *Af*, 1 ND)	1	5.0 (0.2-22.3)	1.0	0	0	0.453
	19 *Af*	1	5.3 (0.3-23.3)		0	0	
Landfill I (trapping in September)	11 (5 Mg, 6 *Af*)	0	0	0.128	4	36.4 (12.8-66.4)	0.01
	6 *Af*	0	0		2	33.3 (6.0-73.8)	
	5 Mg	0	0		2	40 (7.3-81.8)	
Landfill II (trapping in September)	24 (2 Mg, 7 *Af*, 12 *Aa*, 2 As*,* 1 ND)	5	20.8 (8.1-40.3)	0.546	0	0	0.023
	7 *Af*	1	14.3 (0.7-53.0)		0	0	
	12 *Aa*	3	25 (6.8-54.1)		0	0	
	2 Mg	0	0		0	0	
Landfill III (trapping in September)	19 (1 Mg, 6 *Af*, 3 *Aa*, 3 As, *1 Au,* 5 ND)	0	0	1.0	0	0	0.023
	6 *Af*	0	0		0	0	
	3 *Aa*	0	0		0	0	
Chernechyi Lis (Monk's Forest)	42 (23 Mg, 11 *Af*, 4 *Aa*, 1 Mm, 3 ND)	3	7.1 (1.8-18.2)	nd	10	23.8 (12.8-38.4)	nd
	11 *Af*	0	0		1	9.1 (0.5-37.3)	
	4 *Aa*	0	0		0	0	
	23 Mg	3	13.0 (3.4-31.5)		9	39.1 (21.1-59.8)	
trapping in July	25 (11 Mg, 8 *Af*, 4 *Aa*, 1 Mm, 1 ND)	2	8.0 (1.4-24.0)	nd	4	16.0 (5.3-34.2)	nd
trapping in October	17 (12 Mg, 3 *Af*, 2 ND)	1	5.9 (0.3-25.8)	nd	6	35.3 (15.7-59.5)	nd
Total	116	9	7.8 (3.9-13.8)	nd	14	12.1 (7.0-19.0)	nd

**Table 9:** The prevalence of parasites and bacteria in muroid rodents using PCR, with subsequent confirmation by sequencing depending on the place of capture (*Mg* – *Myodes glareolus*, *Af* – *Apodemus flavicollis*, *Aa* – *Apodemus agrarius*, *As* – *Apodemus sylvaticus*, *Au* – *Apodemus uralensis*, *Mm* – *Mus musculus*, ND – not identified).

Place of rodent trapping	n animals	*T. grosi*			*Hepatozoon* spp.			*Mycoplasma*-like (mollicutes) spp.			*Bartonella* spp.		
		n positive animals	in % (95% CI)	*p* value	n positive animals	in % (95% CI)	*p* value	n positive animals	in % (95% CI)	*p* value	n positive animals	in % (95% CI)	*p* value
Landfill II (trapping in September)	12 (4 *Af*, 6 *Aa*, 2 As)	5	41.7 (17.2-69.8)	0.055	2	16.7 (2.9-45.1)	0.495	4	33.3 (11.6-62.3)	0.117	6	50.0 (23.4-76.6)	0.042
	4 *Af*	1	25.0 (1.3-75.8)		0	0		1	25.0 (1.3-75.8)		4	100.0 (47.3-100.0)	
	6 *Aa*	4	66.7 (26.2-94.0)		0	0		3	50.0 (14.7-85.3)		1	16.7 (0.8-59.1)	
Landfill III (trapping in September)	12 (1 Mg, 5 *Af*, 1 *Aa*, 3 As, 2 ND)	0	0	1.0	0	0	1.0	0	0	1.0	12	100.0 (77.9-100.0)	1.0
	5 *Af*	0	0		0	0		0	0		5	100.0 (54.9-100.0)	
Chernechyi Lis (Monk's Forest)	8 (4 Mg, 2 *Af*, 1 *Aa*, 1 ND	0	0	nd	0	0	nd	0	0	nd	8	100.0 (68.8-100.0)	nd
	4 Mg	0	0		0	0		0	0		4	100.0 (47.3-100.0)	
Total	32	5	15.6 (6.0-31.3)		2	6.3 (1.1-19.2)		4	12.5 (4.1-27.5)		26	81.3 (65.0-92.0)

**Table 10:** *p* value based on the data from all 116 muroid according to the parameters (t-test and one-way ANOVA)

Microscopy		
Parameter	*Trypanosoma* spp.	*Hepatozoon* spp.
Specie of muroids	0.763	<0.001
Female/Male	0.248	0.614
Weight of rodents	0.396	0.074
Place of muroid trapping	0.075	<0.001
Month of trapping	0.800	0.362

PCR and subsequent sequencing				
Parameter	*T. grosi*	*Hepatozoon* spp.	Mollicutes	*Bartonella* spp.
Specie of muroids	0.009	0.371	0.620	<0.001
Female/Male	0.018	0.935	0.002	0.066
Weight of rodents	0.943	0.002	0.028	0.802
Place of muroid trapping	0.005	0.477	0.019	<0.001
Month of trapping	0.022	0.083	0.043	0.011

### 3.6 Parasites and bacteria according to the different months of trapping and sampling

The prevalence of parasites and bacteria among animals trapped in July, September and October is shown in Table 5, Table 6, Table 7, Table 8, Table 9. A statistically significant association (Table 10) was identified based on the PCR and subsequent sequencing results for *Trypanosoma grosi* (*p* = 0.022), Mollicutes (*p* = 0.043) and *Bartonella* spp. (*p* = 0.011).

## 4 Discussion

Despite numerous studies that have been carried out since the ChNPP disaster (Beresford et al., 2022; Lypska et al., 2022, 2023; Tintori et al., 2024; Riabchenko et al., 2025), there is still no conclusive evidence regarding the effects of long-term radioactive contamination on either macroorganisms in the ChEZ, such as muroid rodents as model species, or on the pathogens they may carry. Across different parts of the world, Muroidea rodents as well as other small mammals harbour haemoparasites such as *Hepatozoon* spp., *Babesia* spp. and *Trypanosoma* spp., while prevalent bacterial communities include *Bartonella* spp., *Mycoplasma* spp., *Borrelia* spp., *Rickettsia* spp. and Anaplasmataceae (Anderson, 1990; Kim et al., 2016; Kamani et al., 2018; Goodrich et al., 2020; Perles et al., 2023; Erickson et al., 2025). Their prevalence patterns, dominant genera and species diversity, however, differ between geographic regions. For mice and voles in Europe, infections are mainly caused by *Hepatozoon* spp. and *Babesia* spp., with *Trypanosoma* spp. occurring less frequently, while among bacteria *Bartonella* spp. prevail (Molyneux, 1970;Healing, 1981; Karbowiak and Wita, 2004; Criado-Fornelio et al., 2006; Welc-Falęciak et al., 2008; Karbowiak et al., 2009; Bajer et al., 2014; Kallio et al., 2014; Hamšíková et al., 2016a; Divari et al., 2021; Ji et al., 2021; Špitalská et al., 2022; Ferrari et al., 2022; Tołkacz et al., 2023). Differences in the reported prevalence of the pathogens are related to the seasonality, particularly for parasites whose vectors are arthropods, partly to the methods of research used, since some studies provide data based only on microscopy, whereas others rely solely on molecular techniques. As a result of our research, we were able to not only microscopically detect *Trypanosoma* spp. and *Hepatozoon* spp. but also confirm their presence using molecular methods and subsequent sequencing. In addition, the presence of bacteria in muroids, including *Bartonella* spp. and mollicutes (*Mycoplasma-*like spp.), was identified, and their genotypes were determined according to animal species and locations with different level of radionuclides, including control trapping site outside ChEZ.

Our study is the first known investigation of muroids in Ukraine in which *T. grosi* was microscopically detected, confirmed by molecular methods, and characterised using phylogenetic analysis. Previous research has shown that social vole (*Microtus socialis* (Pallas, 1773)) trapped near Askania Nova in Ukraine can harbour bloodstream trypomastigotes, which were later identified morphologically as *Trypanosoma microti* Laveran and Pettit, 1909; Wita et al. (2003); Karbowiak et al. (2004). No other reports of *Trypanosoma* detection among mammals in Ukraine have been documented yet. The initial results of our microscopic investigations were described previously; however, the species identity could not be determined at that time (Semenko et al., 2020). Our morphometric study of *T*. *grosi* (Table 3) showed that the measurements in *A*. *agrarius* corresponded to those reported by Sato et al. (2003) and Karbowiak et al. (2009).

Identification of *Herpetosoma* trypanosomes within this group based solely on morphological characteristics and biological criteria is difficult (Noyes et al., 2002; Sato et al., 2005; Zechmeisterová et al., 2021). The 18S ribosomal RNA gene is one of the most variable for *Herpetosoma* trypanosomes and considered as good candidate for strain and species differentiation (Fernandes et al., 1993;Sato et al., 2005; Guan et al., 2011; Vandersea et al., 2015; Dario et al., 2017; Winterhoff et al., 2020). This region enabled the differentiation among *Trypanosoma evotomys* Hadwen, 1912 and *T. grosi*, similarly from *T. microti*, *Trypanosoma musculi* Kendall, 1906 and *Trypanosoma lewisi* (Kent, 1880; Noyes et al., 2002). At the same time, the difference between the genotypes of different *Herpetosoma* species may not be very significant (Mafie et al., 2019). For example, between *T. lewisi* and *T. musculi* there is only one single base change at position 1321 of their 2219-bp SSU rDNA (Hoare, 1972; Haag et al., 1998; Sato et al., 2007). Considering the length of the genotype we obtained (912 bp), this limited resolving power of the 18S rRNA to distinguish closely related species or lineages may account for the low bootstrap support at internal nodes within the *T. lewisi* species complex in our phylogenetic analysis. However, the placement of our genotype within the *T. grosi* clade is supported by 87% bootstrap and is consistent with previous molecular characterisations of this species in rodents of the genus *Apodemus*.

Previously in Ukraine, *Hepatozoon* spp. was detected in *Ixodes* sp. ticks (Hamel et al., 2013), in dogs which had been relocated to Poland from Ukraine (Bajer et al., 2023), as well as in the puppy with clinical signs described for the first time (Galat et al., 2026). In the last two of these cases, however, the agent identified was *Hepatozoon canis* (James, 1905). Our preliminary studies had reported the presence of *Hepatozoon* spp. in mice and voles based on the results of microscopy (Lypska et al., 2023). Two genotypes isolated from the spleen of *A. flavicollis* in the present study are reported here for the first time not only from the ChEZ, but from Ukraine as whole. Phylogenetic analysis placed these genotypes (Fig. 5) in a strongly supported clade together with two previously deposited SK3 genotypes obtained from the same rodent specie in Slovakia (AN KU597250 and PP420938). These results lend further support to the earlier hypothesis of Hamšíková et al. (2016b) and Ganzinelli et al., 2024 regarding the occurrence of this lineage in Europe and extend its known geographical range. In addition, the genotypes KT274177 and KT274178 (Uiterwijk et al., 2023) are also placed within this clade. However, the latter genotypes, isolated not only from *A. flavicollis* but also from *A. sylvaticus* in Croatia, were most likely misidentified by the original authors as *Hepatozoon ayorgbor* (Ganzinelli et al., 2024; Uiterwijk et al., 2023). The apparent misidentification of the rodent-derived genotypes as reptile genotypes may be explained by the conservative nature of the 18S rRNA locus, which is too conserved for the reliable delineation of closely related lineages. According to our phylogenetic analyses, the sequences obtained from rodents of the genus *Apodemus* form a separate, well-supported clade that is sister to, but distinct from, the *H. ayorgbor* and *H. ophisauri* cluster (Sloboda et al., 2008; Abdel-Baki et al., 2014; Jegede et al., 2018). Consequently, the possibility that genotypes obtained in our research correspond to *Hepatozoon sylvatici* Coles, 1914 cannot be excluded (Ganzinelli et al., 2024), particularly given that this specie has only been described morphologically and genetic data are not yet available. Because no parasites were detected microscopically in blood films from *A. flavicollis* harbouring these genotypes in our study, this hypothesis cannot currently be confirmed or refuted and should be addressed in future studies. Similarly, in the absence of a morphological description of the parasites represented by these genotypes, both transmission routes and probable vectors remain unknown (Hamšíková et al., 2016b; Ganzinelli et al., 2024).

In our phylogenetic analysis of *Hepatozoon* spp., the bootstrap support for both lineages, the *Apodemus*-associated and the *M. glareolus* lineage, reached 98%, while they remained clearly distinct. The separation of these lineages is further supported by the prevalence data for *H. erhardovae,* which, together with types SK1 and SK2, forms the *M. glareolus* lineage (Hamšíková et al., 2016b; Ganzinelli et al., 2024), being detected in 43% of voles of the genus *Clethrionomys* and 2% in *Microtus agrestis*, whereas it was not detected in mice of the genus *Apodemus* (Laakkonen et al., 2001).

Our study represents the first Mollicutes (*Mycoplasma*-like spp.) identification in Ukraine. Previously, *Mycoplasma anserisalpingitidis* Volokhov et al., 2020 was reported in geese, *Mycoplasma agalactiae* (Wroblewski, 1931; Gupta et al. (2018) in small ruminants, and *Mycoplasma haemocanis*, formerly *Haemobartonella canis* (Kikuth, 1928) in dogs (Grózner et al., 2021; Bohach et al., 2021, 2022; Adaszek et al., 2024). The data we obtained indicate a total prevalence of Mollicutes at 12.5%, which is significantly lower than the results obtained in Poland (68.3%) and then that of *Bartonella* spp. (81.3%) prevalence from our research. According to the results of previous studies on the territory of the ChEZ, reported prevalence was 38.9% (Szewczyk et al., 2021). With that, *B*. *taylorii* and *B*. *grahamii* were among the genotypes detected.

Our results indicated a statistically significant effect of month of capture on the prevalence of *T*. *grosi*, Mollicutes and *Bartonella* spp. in muroids even though our sampling months were limited to July, September and October. This observation is consistent with findings from other studies (Karbowiak and Siński, 1996; Karbowiak and Wita, 2001; Bajer et al., 2001; Pawelczyk et al., 2004; Karbowiak et al., 2009). Nevertheless, in our dataset the pattern was evident only in PCR output and was not mirrored in the microscopic studies.

Our results revealed statistically significant sex-related differences in the prevalence of *Trypanosoma* spp. and *Mycoplasma*-like bacteria. A similar pattern to have been observed in the study by Fabbri et al. (2026), with higher infestation rates in males than in females, which is broadly consistent with our findings. This may be related to hormonal and immunological variation between males and females (Greenblatt and Rosenstreich, 1984), behavioural and social-structural contrasts (Fabbri et al., 2026), as well as unequal exposure to vectors (Smith et al., 2005).

In our study, the level of radioactive contamination differed both in relation to the measured radioactive pollution of the different places of trapping muroids, including landfills I, II and III, and with respect to different species of muroids. Although this indicator has shown a declining trend since the accident, it remains consistently high in the most contaminated areas, such as Rudyi Lis (Red Forest) (Beresford et al., 2022). Compared with the Rudyi Lis, the levels of radionuclides, particularly ^137^Cs, ^90^Sr, ^241^Am, were lower at the landfills I, II, III on the coastline of the ChNPP cooling pond, where animal trapping was also conducted. At the same time, the radiation exposures on animals according to the data Beresford et al. (2020) depends on both external and internal exposure to the total dose rate and varies in their relative contribution in different species of animals including muroids, for example, feeding preferences and adaptations to living conditions. Based on one of the radionuclides, ^137^Cs, the absorbed dose was determined across the different animal species examined. The highest value was observed in *A*. *flavicollis*, followed by slightly lower value in *A*. *agrarius*, with *M*. *glareolus*, showing the lowest value. Interestingly, *T*. *grosi* was detected in 5.3% of *A. flavicollis* in Rudyi Lis and 14.3% in landfill II while in control trapping place all animals were negative with the help of microscopy. In contrast, among *M*. *glareolus* from landfill I and the control group of animals from Chernechyi Lis, almost no differences were detected for the prevalence of *Hepatozoon* spp. Moreover, among rodents in the control group, only one *Bartonella* spp. genotype (PZ020206) was isolated, whereas all other pathogen genotypes were identified in muroids from the ChEZ. More detailed research is needed to identify the synergistic effects of both radionuclides and various pathogens on animals in different place of ChEZ including the dependence of these data on the species characteristics of animals and seasonal dependence of the presence of pathogens and their vectors.

## 5 Conclusions

Our study adds new information on the prevalence of muroid parasites and bacteria in Ukraine within the ChEZ under varying levels of radioactive contamination and in comparison with a control group of animals. Thus, for the first time, we detected the presence of *T*. *grosi* in *A. agrarius, Hepatozoon* spp. SK3-type in *A. flavicollis*, Mollicutes and *Bartonella* spp. genotypes. These results represent a logical continuation of numerous studies by other groups of scientists on the effects of acute and then chronic radioactive contamination on the host organism, which have been ongoing for the past 40 years after one of the largest nuclear power plant accidents in the world.

## Ethical statement

The study described in this article was based on muroid tissues that had not been involved in prior assessment of radioactive contamination in animals from Chornobyl Exclusion Zone, conducted under a programme of the Institute for Nuclear Research of the National Academy of Sciences of Ukraine. It was approved by the Bioethics Commission of the Institute for Nuclear Research of the National Academy of Sciences in Ukraine and was carried out in accordance with the Ukrainian Law ‘On the Protection of Animals against Cruelty’ (No. 3447-IV, 2017).

## Funding

The authors received no financial support for the research, authorship, and publication of this paper.

## CRediT authorship contribution statement

**Vladyslava Storozhuk:** Data curation, Formal analysis, Investigation, Writing – original draft. **Hans-Peter Fuehrer:** Methodology, Project administration, Supervision, Writing – review & editing. **Bita Shahi Barogh:** Investigation, Writing – review & editing. **Barbara Eigner:** Investigation, Writing – review & editing. **Alla Lypska:** Investigation, Writing – review & editing. **Olena Burdo:** Investigation, Writing – review & editing. **Denys Vyshnevskyi:** Investigation, Writing – review & editing. **Olena Semenko:** Investigation, Writing – review & editing. **Caroline F. Frey:** Project administration, Supervision, Writing – review & editing. **Maryna Galat:** Conceptualization, Investigation, Methodology, Project administration, Supervision, Writing – original draft, Writing – review & editing.

## Declaration of competing interest

The authors declare that they have no conflict of interest.
