# Supplementary material for: Blood parasites and bacteria of Muroidea rodents of the Chornobyl Exclusion Zone
Source: Int J Parasitol Parasites Wildl. 2026 Jun 12;30:101250. doi: 10.1016/j.ijppaw.2026.101250 (PMC13312017; doi:10.1016/j.ijppaw.2026.101250)
Supplement: Multimedia component 1 [file mmc1.docx]

Table 1. The prevalence of parasites and bacteria in muroid rodents of various species using microscopy and PCR, with subsequent pathogen confirmation by sequencing

| Rodent specie | Microscopy | | | | | PCR and subsequent sequencing of positive samples | | | | | | | | |
| --- | --- | --- | --- | --- | --- | --- | --- | --- | --- | --- | --- | --- | --- | --- |
|  | n animals | *Trypanosoma* spp. | | *Hepatozoon* spp. | | n animals | *Trypanosoma* sp./*T. grosi* | | *Hepatozoon* spp. | | *Mycoplasma*-like spp. | | *Bartonella* spp. | |
|  |  | n positive animals | % (95% CI) | n positive animals | % (95% CI) |  | n positive animals | % (95% CI) | n positive animals | % (95% CI) | n positive animals | % (95% CI) | n positive animals | % (95% CI) |
| *Myodes glareolus* | 31 | 3 | 9.7 (2.5-24.1)^1^ | 11 | 35.5 (20.3-53.3)^2^ | 5 | 0 | 0 | 0 | 0 | 0 | 0 | 5 | 100.0 (54.9-100.0) |
| trapping in July | 11 | 2 | 18.2 (3.2-48.3)^3^ | 3 | 27.3-(7.5-57.8)^4^ | 4 | 0 | 0 | 0 | 0 | 0 | 0 | 4 | 100.0 (47.3-100.0) |
| trapping in September | 8 | 0 | 0^3^ | 2 | 25.0 (4.4-61.2)^4^ | 1 | 0 | 0 | 0 | 0 | 0 | 0 | 1 | 100.0 (5.0-100.0) |
| trapping in October | 12 | 1 | 8.3 (0.4-34.8)^3^ | 6 | 50.0 (23.4-76.6)^4^ | 0 | 0 | 0 | 0 | 0 | 0 | 0 | 0 | 0 |
| female | 13 | 3 | 23.1 (6.2-50.9)^5^ | 4 | 30.8 (10.6-58.7)^6^ | 4 | 0 | 0 | 0 | 0 | 0 | 0 | 4 | 100.0 (47.3-100.0) |
| male | 17 | 0 | 0^5^ | 7 | 41.2 (20.1-65.0)^6^ | 1 | 0 | 0 | 0 | 0 | 0 | 0 | 1 | 100.0 (5.0-100.0) |
| up to 15 g | 2 | 1 | 50 (2.5-97.5)^7^ | 1 | 50 (2.5-97.5)^8^ | 0 | 0 | 0 | 0 | 0 | 0 | 0 | 0 | 0 |
| 15-21 g | 17 | 0 | 0^7^ | 4 | 23.5 (8.0-47.5)^8^ | 1 | 0 | 0 | 0 | 0 | 0 | 0 | 1 | 100.0 (5.0-100.0) |
| 21-27 g | 11 | 2 | 18.2 (3.2-48.3)^7^ | 6 | 54.5 (25.9-81.0)^8^ | 4 | 0 | 0 | 0 | 0 | 0 | 0 | 4 | 100.0 (47.3-100.0) |
| *Apodemus agrarius* | 19 | 3 | 15.8 (4.2-37.2)^1^ | 0 | 0^2^ | 8 | 4 | 50.0 (18.4-81.6) | 0 | 0 | 2 | 25.0 (4.4-61.2) | 3 | 37.5 (10.6-72.2) |
| trapping in July | 4 | 0 | 0^9^ | 0 | 0 | 1 | 0 | 0 | 0 | 0 | 0 | 0 | 1 | 100.0 (5.0-100.0) |
| trapping in September | 14 | 3 | 21.4 (5.8-48.0)^9^ | 0 | 0 | 7 | 4 | 57.1 (21.6-87.7) | 0 | 0 | 0 | 0 | 2 | 28.6 (5.1-67.0) |
| trapping in October | 1 | 0 | 0^9^ | 0 | 0 | 0 | 0 | 0 | 0 | 0 | 0 | 0 | 0 | 0 |
| female | 8 | 0 | 0^10^ | 0 | 0 | 2 | 1 | 50.0 (2.5-97.5) | 0 | 0 | 0 | 0 | 0 | 0 |
| male | 11 | 3 | 27.3 (7.5-57.8)^10^ | 0 | 0 | 6 | 3 | 50.0 (14.7-85.3) | 0 | 0 | 2 | 33.3 (6.0-73.8) | 3 | 50.0 (14.7-81.3) |
| up to 15 g | 10 | 2 | 20.0 (3.5-52.0)^11^ | 0 | 0 | 4 | 2 | 50.0 (9.4-90.6) | 0 | 0 | 2 | 50.0 (9.4-90.6) | 1 | 25.0 (1.3-75.8) |
| 15-21 g | 3 | 1 | 33.3 (1.7-86.8)^11^ | 0 | 0 | 2 | 1 | 50.0 (2.5-97.5) | 0 | 0 | 0 | 0 | 1 | 50.0 (2.5-97.5) |
| 21-27 g | 6 | 0 | 0^11^ | 0 | 0 | 2 | 1 | 50.0 (2.5-97.5) | 0 | 0 | 0 | 0 | 1 | 50.0 (2.5-97.5) |
| *Apodemus flavicollis* | 49 | 2 | 4.1 (0.7-12.8)^1^ | 3 | 6.1 (1.6-15.8)^2^ | 11 | 1 | 9.1 (0.5-37.3) | 2 | 18.2 (3.2-48.3) | 2 | 18.2 (3.2-48.3) | 10 | 90.9 (62.7-99.6) |
| trapping in July | 8 | 0 | 0^12^ | 1 | 12.5 (0.6-48.0) ^13^ | 2 | 0 | 0 | 0 | 0 | 0 | 0 | 2 | 100.0 (22.4-100.0) |
| trapping in September | 20 | 1 | 5.0 (0.2-22.3)^12^ | 2 | 10.0 (1.7-29.3) ^13^ | 9 | 1 | 11.1 (0.6-48.0) | 2 | 22.2 (3.9-56.2) | 2 | 22.2 (3.9-56.2) | 8 | 88.9 (56.1-99.4) |
| trapping in October | 21 | 1 | 4.8 (0.2-21.3)^12^ | 0 | 0^13^ | 0 | 0 | 0 | 0 | 0 | 0 | 0 | 0 | 0 |
| female | 30 | 1 | 3.3 (0.2-15.4)^14^ | 2 | 6.7 (1.1-20.3) ^15^ | 8 | 0 | 0 | 1 | 12.5 (0.6-48.0) | 0 | 0 | 7 | 87.5 (52.0-99.4) |
| male | 18 | 1 | 5.6 (0.3-24.5)^14^ | 1 | 5.6 (0.3-24.5) ^15^ | 2 | 0 | 0 | 1 | 50.0 (2.5-97.5) | 1 | 50.0 (2.5-97.5) | 2 | 100.0 (22.4-100.0) |
| nd | 1 | 0 | 0 | 0 | 0 | 1 | 1 | 100 (5.0-100.0) | 0 | 0 | 1 | 100 (5.0-100.0) | 0 | 0 |
| up to 15 g | 8 | 1 | 12.5 (0.6-48.0)^16^ | 0 | 0^17^ | 2 | 0 | 0 | 1 | 50.0 (2.5-97.5) | 0 | 0 | 1 | 50.0 (2.5-97.5) |
| 15-21 g | 12 | 0 | 0^16^ | 2 | 16.7 (2.9-49.1) ^17^ | 5 | 1 | 20.0 (1.1-70.1) | 0 | 0 | 1 | 20.0 (1.1-70.1) | 4 | 80.0 (29.9-98.9) |
| 21-27 g | 12 | 1 | 8.3 (0.4-34.8)^16^ | 1 | 8.3 (0.4-34.8) ^17^ | 3 | 0 | 0 | 0 | 0 | 0 | 0 | 3 | 100.0 (36.8-100.0) |
| over 27 g | 17 | 0 | 0^16^ | 0 | 0^17^ | 1 | 0 | 0 | 1 | 100.0 (5.0-100.0) | 0 | 0 | 1 | 100.0 (5.0-100.0 |
| *Apodemus sylvaticus* | 5 | 0 | 0^1^ | 0 | 0^2^ | 5 | 0 | 0 | 0 | 0 | 0 | 0 | 5 | 100.0 (54.9-100.0) |
| trapping in July | 0 | 0 | 0 | 0 | 0 | 0 | 0 | 0 | 0 | 0 | 0 | 0 | 0 | 0 |
| trapping in September | 5 | 0 | 0 | 0 | 0 | 5 | 0 | 0 | 0 | 0 | 0 | 0 | 5 | 100.0 (54.9-100.0) |
| trapping in October | 0 | 0 | 0 | 0 | 0 | 0 | 0 | 0 | 0 | 0 | 0 | 0 | 0 | 0 |
| female | 3 | 0 | 0 | 0 | 0 | 3 | 0 | 0 | 0 | 0 | 0 | 0 | 2 | 66.7 (13.2-98.3) |
| male | 2 | 0 | 0 | 0 | 0 | 2 | 0 | 0 | 0 | 0 | 0 | 0 | 2 | 100.0 (22.4-100.0) |
| up to 15 g | 3 | 0 | 0 | 0 | 0 | 3 | 0 | 0 | 0 | 0 | 0 | 0 | 3 | 100.0 (36.8-100.0) |
| 15-21 g | 2 | 0 | 0 | 0 | 0 | 2 | 0 | 0 | 0 | 0 | 0 | 0 | 1 | 50.0 (2.5-97.5) |
| *Apodemus uralensis* | 1 | 0 | 0^1^ | 0 | 0^2^ | 0 | 0 | 0 | 0 | 0 | 0 | 0 | 0 | 0 |
| *Mus musculus* | 1 | 0 | 0 | 0 | 0 | 0 | 0 | 0 | 0 | 0 | 0 | 0 | 0 | 0 |
| ND | 10 | 1 | 10.0 (0.5-40.4)^1^ | 0 | 0^2^ | 3 | 0 | 0 | 0 | 0 | 0 | 0 | 3 | 100.0 (36.8-100.0) |
| Total | 116 | 9 | 7.8 (3.9-13.8) | 14 | 12.1 (7.0-19.0) | 32 | 5 | 15.6 (6.0-31.3) | 2 | 6.3 (1.1-19.2) | 4 | 12.5 (4.1-27.5) | 26 | 81.3 (65.0-92.0) |

Explanations: ANOVA: ^1^p=0.763, ^2^p=0.001, ^3^p=0.434, ^4^p=0.430, ^7^p=0.088, ^8^p=0.343, ^9^p=0.574, ^11^p=0.420,^12^p=0.825, ^13^p=0.306, ^16^p=0.375, ^17^p=0.269; t-test: ^5^p=0.046, ^6^p=0.482, ^10^p=0.120, ^14^p<0.001, ^15^p=0.958

Table 2. The prevalence of parasites and bacteria in muroid rodents using microscopy and PCR, with subsequent pathogen confirmation by sequencing depending on the place of capture (*Mg* – *Myodes glareolus*, *Af* – *Apodemus flavicollis*, *Aa* – *Apodemus agrarius*, *As* – *Apodemus sylvaticus*, *Au* – *Apodemus uralensis*, *Mm* – *Mus musculus*, ND – not identified)

| Place of rodent trapping | Microscopy | | | | | PCR and subsequent sequencing of positive samples | | | | | | | | |
| --- | --- | --- | --- | --- | --- | --- | --- | --- | --- | --- | --- | --- | --- | --- |
|  | n animals | *Trypanosoma* spp. | | *Hepatozoon* spp. | | n animals | *T.* *grosi* | | *Hepatozoon* spp. | | *Mycoplasma*-like (mollicutes) spp. | | *Bartonella* spp. | |
|  |  | n positive animals | 95 % (CI) | n positive animals | 95 % (CI) |  | n positive animals | 95 % (CI) | n positive animals | 95 % (CI) | n positive animals | 95 % (CI) | n positive animals | 95 % (CI) |
| Rudyi Lis (Red Forest) (trapping in October) | 20  (19 *Af*, 1 ND) | 1 | 5.0 (0.2-22.3)^1^ | 0 | 0^5^ | nd | | | | | | | | |
|  | 19 *Af* | 1 | 5.3 (0.3-23.3) | 0 | 0 | nd | | | | | | | | |
| Landfill I (trapping in September) | 11  (5 *Mg*, 6 *Af*) | 0 | 0^2^ | 4 | 36.4 (12.8-66.4)^6^ | nd | | | | | | | | |
|  | 6 *Af* | 0 | 0 | 2 | 33.3 (6.0-73.8) | nd | | | | | | | | |
|  | 5 *Mg* | 0 | 0 | 2 | 40 (7.3-81.8) | nd | | | | | | | | |
| Landfill II (trapping in September) | 24  (2 *Mg*, 7 *Af*, 12 *Aa*, 2 *As,*  1 ND) | 5 | 20.8 (8.1-40.3)^3^ | 0 | 0^7^ | 12 (4 *Af*, 6 *Aa*, 2 *As*) | 5 | 41.7 (17.2-69.8)^9^ | 2 | 16.7 (2.9-45.1)^10^ | 4 | 33.3 (11.6-62.3)^11^ | 6 | 50.0 (23.4-76.6)^12^ |
|  | 7 *Af* | 1 | 14.3 (0.7-53.0) | 0 | 0 | 4 *Af* | 1 | 25.0 (1.3-75.8) | 0 | 0 | 1 | 25.0 (1.3-75.8) | 4 | 100.0 (47.3-100.0) |
|  | 12 *Aa* | 3 | 25 (6.8-54.1) | 0 | 0 | 6 *Aa* | 4 | 66.7 (26.2-94.0) | 0 | 0 | 3 | 50.0 (14.7-85.3) | 1 | 16.7 (0.8-59.1) |
|  | 2 *Mg* | 0 | 0 | 0 | 0 | nd | nd | nd | nd | nd | nd | nd | nd | nd |
| Landfill III (trapping in September) | 19  (1 *Mg*, 6 *Af*, 3 *Aa*, 3 *As*, *1 Au,*  5 ND) | 0 | 0^4^ | 0 | 0^8^ | 12 (1 *Mg*, 5 *Af*, 1 *Aa*, 3 *As*, 2 ND) | 0 | 0^13^ | 0 | 0^14^ | 0 | 0^15^ | 12 | 100.0 (77.9-100.0)^16^ |
|  | 6 *Af* | 0 | 0 | 0 | 0 | 5 *Af* | 0 | 0 | 0 | 0 | 0 | 0 | 5 | 100.0 (54.9-100.0) |
|  | 3 *Aa* | 0 | 0 | 0 | 0 |  |  |  |  |  |  |  |  |  |
| Chernechyi Lis (Monk’s Forest) | 42  (23 *Mg*, 11 *Af*, 4 *Aa*, 1 *Mm*, 3 ND) | 3 | 7.1 (1.8-18.2) | 10 | 23.8 (12.8-38.4) | 8 (4 *Mg*, 2 *Af*, 1 *Aa*, 1 ND | 0 | 0 | 0 | 0 | 0 | 0 | 8 | 100.0 (68.8-100.0) |
|  | 11 *Af* | 0 | 0 | 1 | 9.1 (0.5-37.3) |  |  |  |  |  |  |  |  |  |
|  | 4 *Aa* | 0 | 0 | 0 | 0 |  |  |  |  |  |  |  |  |  |
|  | 23 *Mg* | 3 | 13.0 (3.4-31.5) | 9 | 39.1 (21.1-59.8) | 4 *Mg* | 0 | 0 | 0 | 0 | 0 | 0 | 4 | 100.0 (47.3-100.0) |
| trapping in July | 25 (11 *Mg*, 8 *Af*, 4 *Aa*, 1 *Mm*, 1 ND) | 2 | 8.0 (1.4-24.0) | 4 | 16.0 (5.3-34.2) | 8 (4 *Mg*, 2 *Af*, 1 *Aa*, 1 ND | 0 | 0 | 0 | 0 | 0 | 0 | 8 | 100.0 (68.8-100.0) |
| trapping in October | 17 (12 *Mg*, 3 *Af*, 2 ND) | 1 | 5.9 (0.3-25.8) | 6 | 35.3 (15.7-59.5) | nd | nd | nd | nd | nd | nd | nd | nd | nd |
| Total | 116 | 9 | 7.8 (3.9-13.8) | 14 | 12.1 (7.0-19.0) | 32 | 5 | 15.6 (6.0-31.3) | 2 | 6.3 (1.1-19.2) | 4 | 12.5 (4.1-27.526) | 26 | 81.3 (65.0-92.0) |

Explanations: Fisher`s exact test: ^1^p=1.0; ^2^p=0.128; ^3^p=0.546; ^4^p=1.0; ^5^p=0.453; ^6^p=0.01; ^7^p=0.023; ^8^p=0.023; ^9^p=0.055; ^10^p=0.495; ^11^p=0.117; ^12^p=0.042; ^13^p=1.0; ^14^p=1.0; ^15^p=1.0; ^16^p=1.0
